# Oncogenic role and drug sensitivity of ETV4 in human tumors: a pan-cancer analysis

**DOI:** 10.3389/fonc.2023.1121258

**Published:** 2023-05-02

**Authors:** Rui Zhang, Yanfang Peng, Zhe Gao, Jing Qian, Kang Yang, Xinfa Wang, Wenjing Lu, Yongjie Zhu, Dezhi Qiu, Tong Jin, Gang Wang, Junping He, Ning Liu

**Affiliations:** ^1^ Department of Neurosurgery, Children’s Hospital of Nanjing Medical University, Nanjing, Jiangsu, China; ^2^ Department of Neurosurgery, The First Affiliated Hospital of Nanjing Medical University, Nanjing, Jiangsu, China; ^3^ Nanjing Drum Tower Hospital, The Affiliated Hospital of Nanjing University Medical School, Nanjing, Jiangsu, China

**Keywords:** ETV4, pancancer, microenvironment, immunity, prognostic, drug sensitivity

## Abstract

**Background:**

Increasing evidence supports a relationship between E twenty-six variant transcription factor 4 (ETV4) and several cancers, but no pan-cancer analysis has been reported.

**Methods:**

The present study surveyed the effects of ETV4 on cancer using RNA sequencing data obtained from The Cancer Genome Atlas and GTEx, and further explored its role in drug sensitivity using data from Cellminer. Differential expression analyses were conducted for multiple cancers using R software. Cox regression and survival analysis were employed to calculate correlations between ETV4 levels and survival outcomes in multiple cancers using the online tool Sangerbox. ETV4 expression was also compared with immunity, heterogeneity, stemness, mismatch repair genes, and DNA methylation among different cancers.

**Results:**

ETV4 was found to be significantly upregulated in 28 tumors. Upregulation of ETV4 was associated with poor overall survival, progression free interval, disease-free-interval, and disease specific survival in several cancer types. Expression of ETV4 was also remarkably correlated with immune cell infiltration, tumor heterogeneity, mismatch repair gene expression, DNA methylation, and tumor stemness. Furthermore, ETV4 expression seemed to affect sensitivity to a number of anticancer drugs.

**Conclusions:**

These results suggest that ETV4 may be useful as a prognostic factor and therapeutic target.

## Introduction

Cancer is a major public health problem that causes remarkable morbidity and mortality. By 2040, the global cancer rate is expected to exceed 29 million cases annually and account for one out of every six deaths (1, 2). As medical technology continuously improves, so too do cancer screening and management. However, only a few malignancies are able to be cured due to the complexity of tumorigenesis, progression, and therapy resistance. Big data may be leveraged to more efficiently investigate the potential molecular mechanisms of tumorigenesis, progression, and guide clinicians to select cancer-sensitivity drugs according to the characteristics of patient’s tumor cells. Pan-cancer analysis is a recent and critical development, useful for uncovering mutations and other genetic abnormalities that are common to many different cancers. The present study investigated the role of E twenty-six variant transcription factor 4 (ETV4) among different cancers *via* pan-cancer analysis.

ETV4 is a member of the E26 transcription factor superfamily, which contains the ETS domain and binds to the core GGA(A/T) sequence (3–5). It plays critical roles in embryonic development, including neurogenesis (4, 6), lung branching (7, 8), spermatogenesis, and limb bud formation (9). Recent studies revealed that ETV4 is aberrantly expressed in many types of tumors, and its overexpression is related to poor prognosis of cancer patients, including breast (10, 11), prostate (12), lung (13), and gastric (10, 14) cancers. Additionally, increasing studies have identified that ETV4 promotes cancer growth, invasion, metastasis, and drug resistance (15). ETV4 may promote cancer metastasis by triggering transcription of ZEB1 and SNAIL1 (16). However, it remains uncertain whether ETV4 contributes to the etiology of various cancers through common molecular processes.

In the current study we investigated ETV4 expression among various tumors and its relationship with patient prognosis, mismatch repair genes, mRNA methylation, immunity, heterogeneity, and stemness using RNA sequencing (RNA-seq) data from The Cancer Genome Atlas (TCGA). We also surveyed the impact of ETV4 levels on drug tolerance and sensitivity using data from CellMiner. Our findings showed that ETV4 may be useful as an oncogenic prognostic factor and therapy target.

## Methods

### Data collection

RNA-seq data and clinical parameters of different cancers and matched normal tissues were collected from TCGA database. The RNA-seq data were then normalized *via* the UCSC Xena tool (https://xena.ucsc.edu/). Genetic profiles of 31 human normal tissues were obtained from GTEx (https://commonfund.nih.gov/GTEx). Duplicated data were excluded.

### Expressional levels and survival outcome analysis of ETV4

The prognostic value of ETV4 in various types of cancers was analyzed using univariate Cox analysis and Kaplan-Meier survival analysis through the online tool Sangerbox (http://vip.sangerbox.com/). Data for expression analysis were from TCGA and GTEx database. Each expression value was transformed *via* log2(x+1) and single cancer with less than three samples was removed. Unpaired Student’s t-Tests were used for differential significance analysis. The clinical parameters used for survival analysis were obtained from TCGA database. Cox regression and Kaplan-Meier survival analysis were used for survival outcome analysis.

### Immunohistochemistry staining

Immunohistochemistry images of ETV4 in tumors and non-tumor specimens were acquired from the Human Protein Atlas (http://www.proteinatlas.org/).

### Immunological correlation analysis

Correlations between ETV4 expression levels and immune checkpoints, ImmuneScore, StromalScore, and ESTIMATEScore were calculated using the online tool Sangerbox with Pearson’s analysis. Data used for the analysis were downloaded from TCGA database.The relationship between ETV4 levels and immune-infiltrating cells (CD4+ T cells, CD8+ T cells, B cells, neutrophils, and dendritic cells) was calculated using the online tool Tumor Immune Estimation Resource (TIMER, https://cistrome.shinyapps.io/timer/) with Pearson’s analysis. A p-value <0.05 was regarded as statistically significant.

### Correlation between ETV4 levels and tumor stemness and heterogeneity

The relationships between ETV4 expression and tumor heterogeneity and stemness were assessed among all TCGA tumor specimens using the online tool Sangerbox with Pearson’s correlation analysis. mRNA and DNA methylation profiles were used to calculate the six stemness scores (ss): RNA expression-based (RNAss), epigenetically regulated RNA expression-based (EREG.EXPss), DNA methylation-based (DNAss), epigenetically regulated DNA methylation-based (EREGMETHss), differentially methylated probes-based (DMPss), and enhancer elements/DNA methylation-based (ENHss) according to previous research (17). These stemness indices range from 0 to 1, where 0 means that the tumor cells are less similar to stem cells, and 1 means that the tumor cells are more similar to stem cells.

### Correlations between ETV4 expression and mismatch repair genes and DNA methyltransferases

Expression data for five MMR genes (PMS2, MLH1, EPCAM, MSH2, and MSH6) were obtained from TCGA database and correlated with ETV4 expression using Spearman’s correlation. A similar method was also employed to assess the correlation between ETV4 levels and four DNA methyltransferases (DNMT1, DNMT2, DNMT3A, DNMT3B).

### Drug sensitivity evaluation

To evaluate the impact of ETV4 levels on drug sensitivity, drug sensitivity and gene expression data were downloaded from the CellMiner database (https://discover.nci.nih.gov/cellminer/) which is a large database for collecting, processing, and integrating molecular data on NCI-60 and other tumor cells. Cell sensitivity to a drug was indicated by the z-score in the compound activity profile; the drug’s anticancer activity increased as the z-score value increased. Data were analyzed using Pearson’s correlation using R (4.2.1) software. Drugs were only included if they were currently in a clinical trial or had been approved by the FDA. A p-value <0.01 was regarded as statistically significant. Findings were visualized using “ggplot2” and “ggpubr” packages.

## Results

### Pan-cancer analysis of ETV4 expression

**Figure 1:**
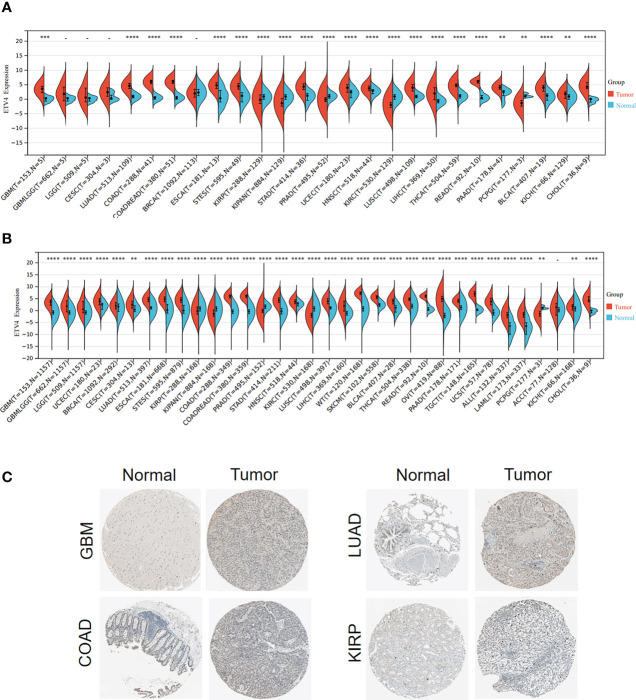
ETV4 expression in various human tumors. **(A)** mRNA levels of ETV4 among different tumors and adjacent normal specimens using TCGA data. **(B)** mRNA levels of ETV4 in tumors and normal tissues using the combined data from TCGA and GTEx. **(C)** Protein expression levels of ETV4 in tumors and their corresponding normal tissues based on The Human Protein Atlas. "-" :no statistic difference **p < 0.01; ***p < 0.001; ****p < 0.0001.

To investigate the differential expression of ETV4 between normal tissues and tumor specimens, mRNA levels were first compared between tissue types using RNA-seq data from TCGA database. As shown in **Figure 1A** and **Supplementary Table 1**, in comparison with normal tissues, ETV4 expression was remarkably elevated in 17 tumors such as glioblastoma multiforme (GBM), lung adenocarcinoma (LUAD), colon adenocarcinoma (COAD), esophageal carcinoma, and colon adenocarcinoma/rectum adenocarcinoma esophageal carcinoma (COADREAD). In some tumors, ETV4 expression was lower than in normal tissues: kidney renal papillary cell carcinoma (KIRP), prostate adenocarcinoma (PRAD), pan-kidney cohort (KIPAN), kidney renal clear cell carcinoma (KIRC), and pheochromocytoma and paraganglioma (PCPG). Because TCGA dataset comprises mainly tumor tissue and very little normal tissue, ETV4 expression was reanalyzed using data from both TCGA and GTEx. As shown in **Figure 1B**, the consolidated result showed that ETV4 was markedly elevated in 28 tumors (including GBM and LUAD) and still downregulated in KIRP, KIPAN, PRAD, KIRC, and PCPG. Similar results were also observed for the immunohistochemical tissue assay (**Figure 1C**). These data reveal that over-expression of ETV4 in most cancer tissues compared to normal tissues.

### Prognostic value of ETV4 among various cancers

**Figure 2:**
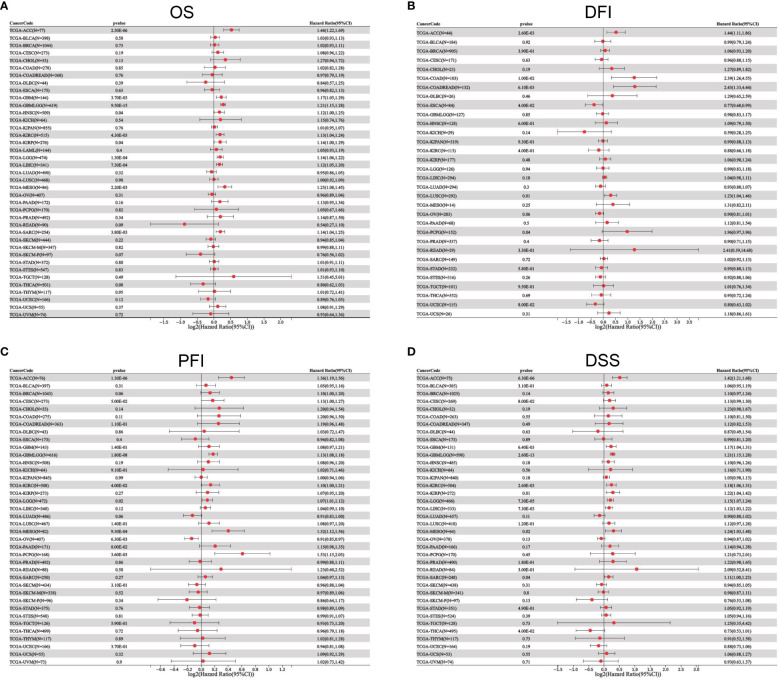
Univariate Cox regression analyses for ETV4 expression in different types of cancers. **(A)** overall survival, **(B)** disease-free interval, **(C)** progression-free interval, **(D)** disease-specific survival. Hazard ratio (HR) values < 1 represent favorable factors, whereas HR values >1 represent risk factors.

**Figure 3:**
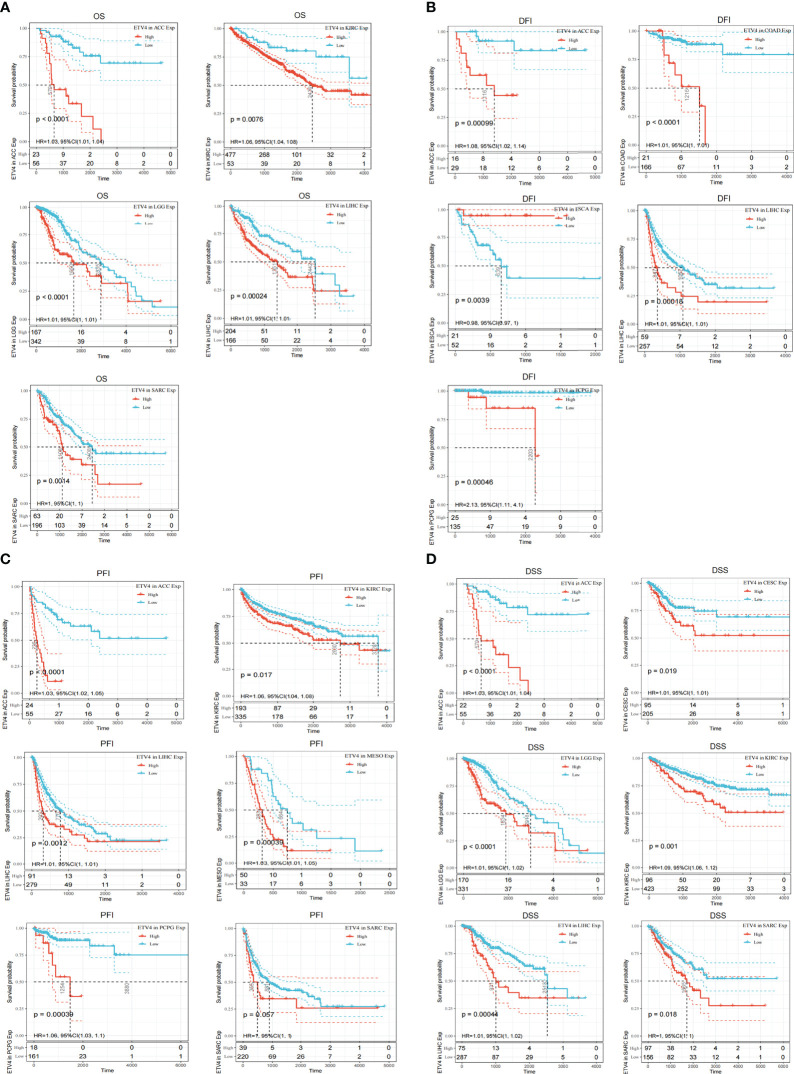
Kaplan-Meier analyses of the correlation between ETV4 expression and overall survival. **(A)**,disease-free interval **(B)**, progression-free interval **(C)**, and disease-specific survival **(D)** in various cancer types.

To better understand the prognostic value of ETV4 in different cancer types, we investigated correlations between ETV4 expression and survival outcomes for each cancer using Cox regression and Kaplan-Meier survival analysis. As seen in **Figures 2A**, **3A**, patients suffering glioma (GBMLGG), brain low grade glioma (LGG), sarcoma, kidney renal papillary cell carcinoma, head and neck squamous cell carcinoma, GBM, KIRC, mesothelioma (MESO), liver hepatocellular carcinoma (LIHC), and adrenocortical carcinoma (ACC) with high expression of ETV4 had worse overall survival. Association analysis of disease-free-interval showed that ETV4 expression was a hazard factor in lung squamous cell carcinoma, COADREAD, COAD, and PCPG, but was a favorable factor in esophageal carcinoma (**Figure 2B** and **Figure 3B**). High expression of ETV4 predicted poorer progression-free interval for GBMLGG, ACC, MESO, PCPG, LGG, and cervical squamous cell carcinoma and endocervical adenocarcinoma (CESC), and better progression-free interval for ovarian serous cystadenocarcinoma (**Figures 2C**, **3C**). High expression of ETV4 was remarkably correlated with poor disease-specific survival in GBMLGG, ACC, LGG, KIRC, GBM, LIHC, KIRP, MESO, and sarcoma, and with improved prognosis in thyroid carcinoma (THCA) (**Figures 2D**, **3D**). The above data indicate that ETV4 may take a pivotal role in the survival of patients with GBMLGG, ACC, LGG, KIRC, GBM, LIHC, KIRP, MESO, sarcoma and THCA.

### Analysis of the relationship between ETV4 expression and immunity

**Figure 4:**
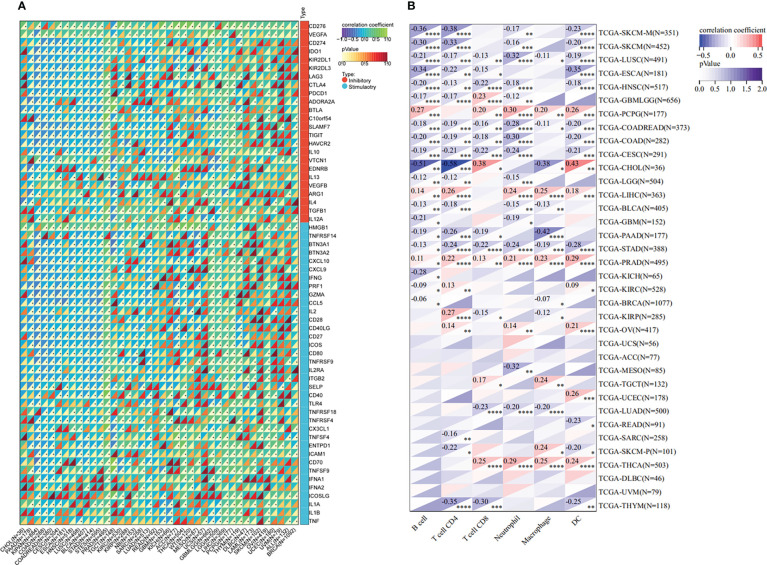
Immunological association analyses. **(A)** The association between ETV4 expression and immune checkpoint genes.The upper triangle of each square represented the magnitude of the p value of the correlation coefficient, and the lower triangle represented the magnitude of correlation test. The star dots in the lower triangle represent that difference is significant. **(B)** The association between ETV4 expression and immune infiltration cells. *p < 0.05; **p < 0.01; ***p < 0.001; ****p < 0.0001.

**Figure 5:**
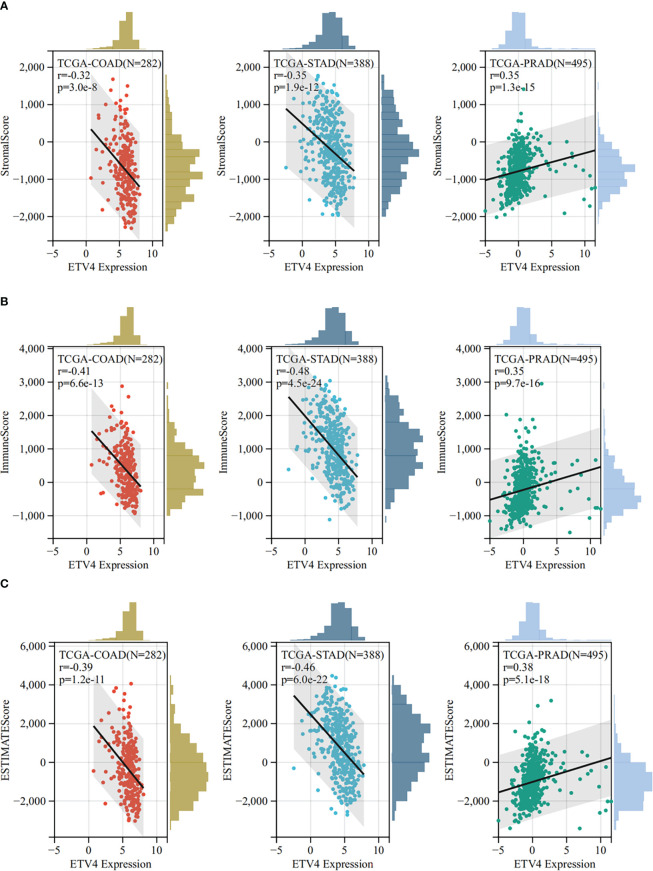
The Sangerbox online tool was used to estimate immune and stromal ratios of various cancers. **(A)** The relationship between ETV4 expression and StromalScore in prostate adenocarcinoma, colon adenocarcinoma, and kidney renal clear cell carcinoma. **(B)** The relationship between ETV4 expression and ImmuneScore in colon adenocarcinoma, prostate adenocarcinoma, and stomach adenocarcinoma. **(C)** The correlation between ETV4 expression and ESTIMATEScore.

The tumor microenvironment plays an important role in tumor initiation, metastasis, progression, and therapeutic outcomes (18, 19) and it has been confirmed that balance of immune checkpoints and immune cells in the TME is critical to suppress tumor development and progression (20, 21). To investigate the function of ETV4 in the tumor microenvironment, we calculated the correlation between ETV4 levels and 60 common immune checkpoint (ICP) genes (36 stimulatory and 24 inhibitory). As shown in **Figure 4A**, the relationship between ETV4 and ICP genes varied among different cancer types. ETV4 expression was positively associated with majority of ICP genes in many cancers, such as ovarian cancer and PCPG, but negatively correlated in KIPAN and COAD.This finding implied that ETV4 might promote tumorigenesis and progression *via* breaking the balance of ICP. Next, relationship between ETV4 levels and immune cell infiltration (comprising B cells, CD8+ T cells, CD4+ T cells, macrophages, neutrophils, and dendritic cells) was also investigated. As shown in **Figure 4B**, ETV4 expression was significantly negative correlated with most of immune cell infiltration in cancers. Additionally, ImmuneScore and StromalScore were also employed to evaluate the relationship between ETV4 expression and immune cell infiltration among cancers. As shown in **Supplementary Figure S1**, ETV4 expression was positively related to StromalScore in GBMLGG, PRAD, thymoma, and PCPG, and negatively related to StromalScore in 19 other tumors. ETV4 expression was associated positively with ImmuneScore and ESTIMATEScore in PRAD and PCPG, and negatively in GBM, LGG, and CESC. The three tumors most strongly correlated with ETV4 expression were PRAD, COAD, stomach adenocarcinoma (STAD) (StromalScore) (**Figure 5A**); PRAD,COAD, STAD (ImmuneScore) (**Figure 5B**); and PRAD, COAD, STAD (ESTIMATEScore) (**Figure 5C**). These results reveal that ETV4 may promote cancer progression *via* regulating immune checkpoint genes expression and immune cells infiltration.

### Analysis of the relationship between ETV4 expression and heterogeneity

**Figure 6:**
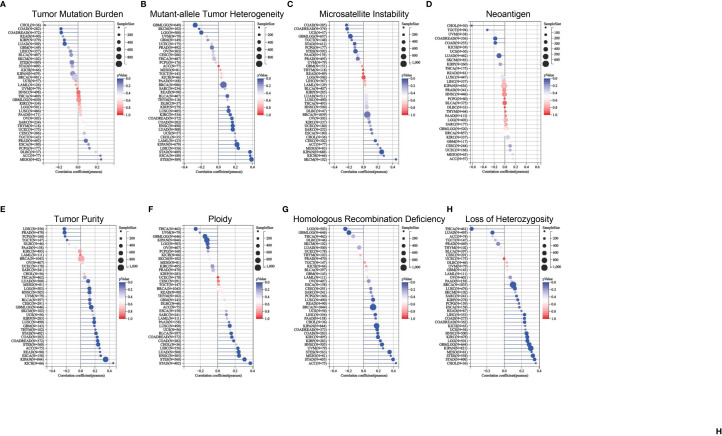
Relationship between ETV4 and tumor heterogeneity in various cancer types. **(A)** tumor mutational burden, **(B)** mutant-allele tumor heterogeneity, **(C)** microsatellite instability, **(D)** neoantigen, **(E)** tumor purity, **(F)** ploidy, **(G)** homologous recombination deficiency, and **(H)** loss of heterozygosity were calculated using data from TCGA datasets.

Heterogeneity is a distinct feature of malignancies. After several divisions and proliferation, their descendants acquire genes or altered molecular biology that lead to further differences in proliferation rates, capacity for invasion and metastasis, or drug resistance. Tumor heterogeneity can be assessed by microsatellite instability (MSI), tumor mutational burden (TMB), homologous recombination deficiency (HRD), tumor purity, neoantigen (NEO), loss of heterozygosity (LOH), tumor ploidy, and mutant-allele tumor heterogeneity (MATH). RNA-seq data from TCGA were used to assess the relationship between ETV4 expression and cancer heterogeneity. As shown in **Figure 6**, ETV4 was positively associated with TMB in MESO and ACC and negatively related to TMB in LUAD, COAD, COADREAD, KIRP, and CHOL. ETV4 was positively associated with MATH in 14 tumors and negatively associated with MATH in GBMLGG, LGG, and skin cutaneous melanoma. ETV4 was positively associated with MSI in CESC, KIPAN, MESO, skin cutaneous melanoma and kidney chromophobe and negatively associated with MSI in COAD, COADREAD, stomach and esophageal carcinoma, STAD and GBMLGG. ETV4 was negatively associated with NEO in LUAD, COAD, COADREAD, testicular germ cell tumors, and CHOL. It was positively associated with tumor purity in 19 tumors and negatively associated with purity in PRAD, LIHC, testicular germ cell tumors, and PCPG. ETV4 was positively associated with ploidy in nine tumors and negatively associated with ploidy in GBMLGG, LGG, KIPAN, THCA, and ovarian serous cystadenocarcinoma. It was related positively to HRD in 14 tumors and negatively in GBMLGG and LGG. ETV4 was positively correlated with LOH in 18 tumors and negatively correlated with LOH in LUAD and THCA. Together these results indicate that ETV4 may accelerate tumor genesis and prognosis by regulating tumor heterogeneity.

### Relationship of ETV4 with tumor stemness

**Figure 7:**
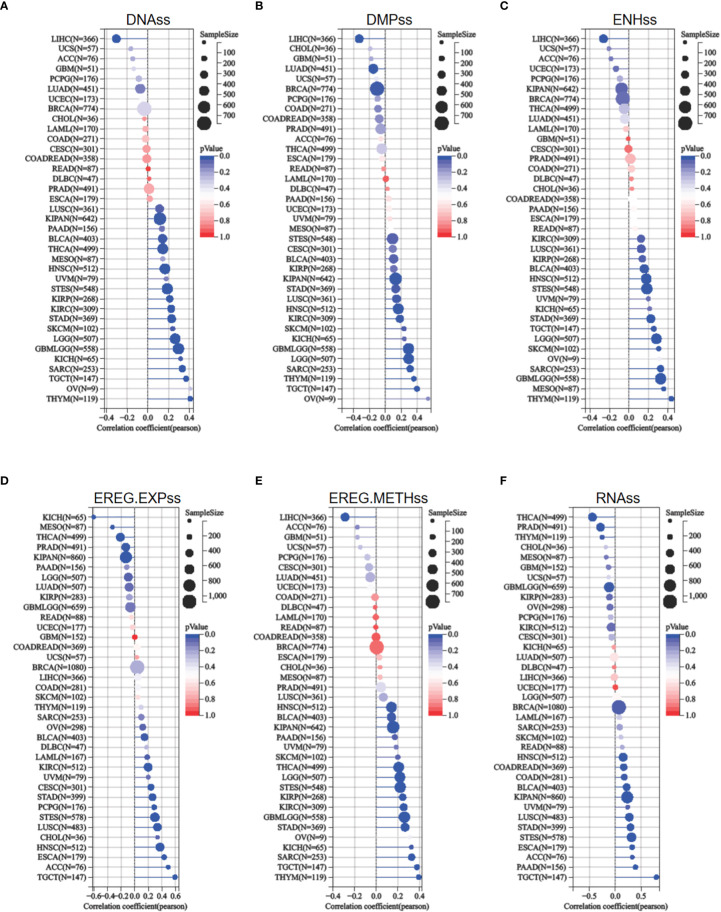
Relationship between ETV4 and cancer stemness in various cancer types. **(A)** DNA expression-based stemness score (DNAss), **(B)** Differentially methylated probes-based stemness score(DMPss), **(C)** Enhancer elements/DNA methylation-based stemness score (ENHss), **(D)** Epigenetically regulated RNA expression-based stemness score (EREG.EXPss), **(E)** Epigenetically regulated DNA methylation-based stemness score(EREG.METHss),and **(F)** RNA expression-based stemness score(RNAss) were calculated using the TCGA datasets.

The acquisition of stem cell-like features is a crucial driver of tumor progression (22). Stemness indices rely on DNA methylation and mRNA expression are calculated to reflect the resemblance between cancer cells and stem cells (17). The indices include RNAss, EREG EXPss, DNAss, EREG METHss, DMPss, and ENHss, each of which varies from zero (least resemblance to stem cells) to one (most resemblance to stem cells). ETV4 expression was compared to stemness indices across various tumor types. As shown in **Figure 7**, ETV4 levels were significantly correlate with RNAss index in 18 cancers (positively associated in 14cancers), EREG EXPss in 20cancers (positively associated in 14cancers), DNAss in 17 cancers (positively associated in16 cancers), EREG METHss in 17 cancers (positively associated in 16 cancers), DMPss in 16 cancers (positively associated in 13 cancers), and ENHss in 15 cancers (positively associated in 14cancers) respectively. These results suggest that ETV4 may be related to the genesis of tumor stemness in most cancers.

### Correlation of ETV4 expression with MMR genes and DNA methyltransferases

**Figure 8:**
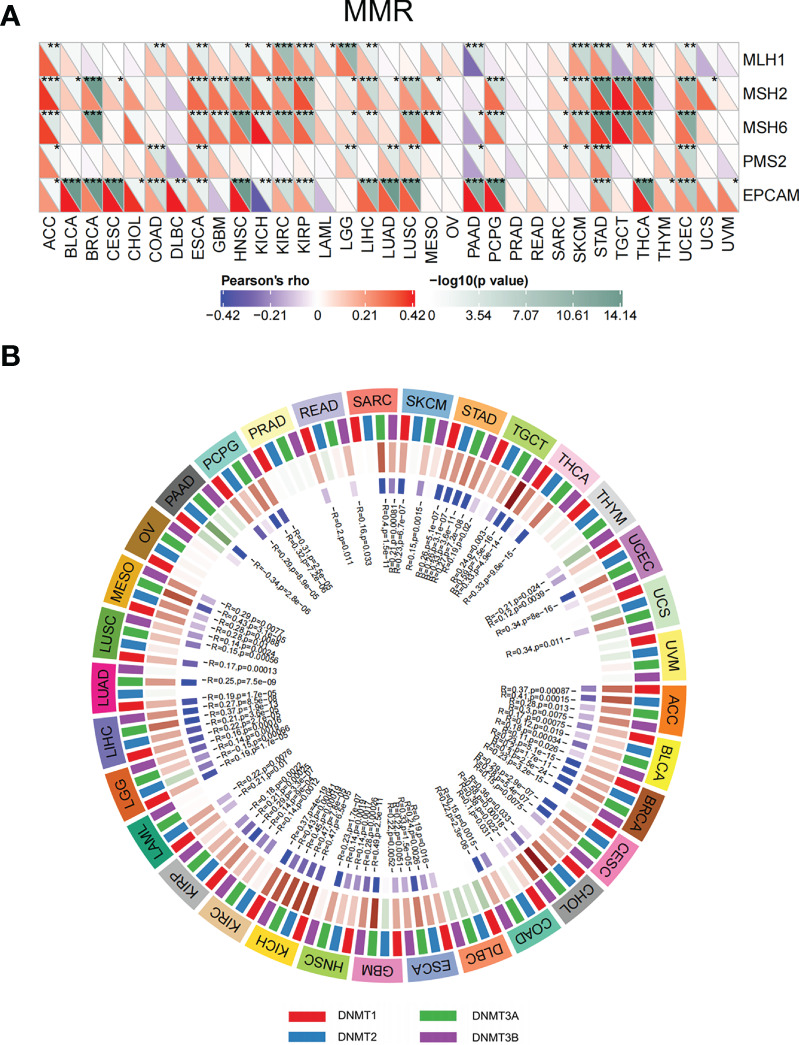
Correlations between ETV4 expression, mismatch repair genes, and DNA methyltransferases among different cancers. **(A)** Relationship between ETV4 expression and mismatch repair genes. The upper triangle of each square represented the magnitude of the p value of the correlation test, and the lower triangle represented the magnitude of correlation coefficient. **(B)** Correlation between ETV4 expression and DNA methyltransferases (Red/green in the third layer referring to correlation coefficient and blue/purple in the forth layer referring to p-value).*p < 0.05, **p < 0.01, ***p < 0.001.

Disorder of MMR genes and DNA methyltransferases plays a pivotal role in tumorigenesis and prognosis (23, 24). To investigate the role of ETV4 in tumorigenesis, ETV4 was correlated with five different MMR genes. As shown in **Figure 8A**, ETV4 was significantly associated with MMR genes in 26 tumors (notably, it was not associated with MMR genes in acute myeloid leukemia, ovarian serous cystadenocarcinoma, PRAD, READ, thymoma, uterine carcinosarcoma, and uveal melanoma). ETV4 was then correlated with four DNA methyltransferases. As shown in **Figure 8B**, ETV4 expression was highly associated with these four methyltransferases in many cancers. These results suggest that ETV4 may promote tumorigenesis and poor prognosis by modulating DNA repair genes and DNA methyltransferases.

### Relationship between ETV4 levels and drug sensitivity

**Figure 9:**
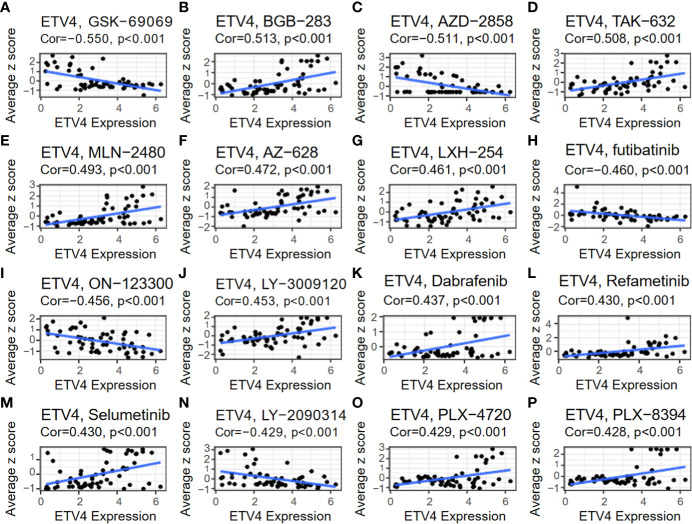
Relationship between ETV4 levels and drug sensitivity. The expression level of ETV4 was correlated with the drug sensitivity of **(A)** GSK-690693, **(B)** BGB-283, **(C)** AZD-2858, **(D)** TAK-632, **(E)** MLN-2480, **(F)** AZ-628, **(G)** LXH-254, **(H)** Futibatinib, **(I)** ON-123300, **(J)** LY-3009120, **(K)** Dabrafenib, **(L)** Refametinib, **(M)** Selumetinib, **(N)** LY-2090314, **(O)** PLX-4720, and **(P)** PLX-8394.

The relationship between ETV4 levels and drug sensitivity was assessed using data from CellMiner. The 16 drugs with the strongest correlations are shown in **Figure 9**. Notably, ETV4 levels were negatively correlated with drug sensitivity of GSK-60903, AZD-2858, Futibatinib, ON-123300, LY-2090314 and positively associated with sensitivity of BGB-283, TAK-632, MLN-2480, AZ-628, LXH-254, LY-3009120, Dabrafenib, Refametinib, Selumetinib, PLX-4720, and PLX-8394. These findings suggest that ETV4 levels can be used for anticancer drug selection.

## Discussion

ETV4 is not only an essential factor for embryonic development (4, 8, 9), but also influences the occurrence and prognosis of many diseases, particularly malignant cancers (10, 11, 13). However, it remains unclear whether ETV4 affects various cancers through common molecular processes.

In the present study the potential function of ETV4 in various cancers was investigated using pan-cancer analysis. Through bioinformatics analyses and immunohistochemistry arrays of tumor tissue, ETV4 expression was found to be elevated in multiple cancers. For many different cancers, patients with higher ETV4 expression had worse disease prognosis. These data were in accordance with previous studies (25–27). Many studies have confirmed that disordered immunity, MMR genes, and DNA methyltransferases contribute to cancer progression and therapeutic outcome (28–30). The present study showed that ETV4 levels were not only positively correlated with ICP genes and immune cell infiltration but also significantly associated with MMR genes and methyltransferases, implying that ETV4 may be useful as an immunotherapy target.

Tumor heterogeneity is defined as the diverse pool of cells within and around tumors that express distinct genotypes and phenotypes and express different signature molecules (31–33). It is a major factor in tumor prognosis, therapy resistance, and recurrence (31, 34). Previous research confirmed that abnormal expression of ETV4 was associated with melanoma heterogeneity (35). To examine whether this effect of ETV4 on tumor heterogeneity is a common phenomenon, we calculated the association between ETV4 and TMB, tumor purity, MSI, NEO, HRD, MATH, LOH, and tumor ploidy among multiple types of cancers. Indeed, the expression of ETV4 was positively associated with tumor heterogeneity in the majority of cancers studied.

Stemness is the ability to self-renew and differentiate from origin cells. Cancer stem cells not only have the ability to self-renew but also to produce heterogeneous tumor cells (36, 37). Accumulating evidence has shown that cancer stem cells play a critical role in tumorigenesis, metastasis (38), drug resistance, and recurrence (39, 40). Tao Zhu and colleagues reported that ETV4 promotes breast cancer cell stemness by activating glycolysis and CXCR4-mediated sonic hedgehog signaling (11). The current study confirmed that ETV4 expression was positively correlated with indices of tumor stemness in most tumor types, suggesting that elevated ETV4 expression may enhance tumor proliferation, metastasis, therapy resistance, and recurrence in general.

Finally, effects of ETV4 on drug sensitivity were investigated. ETV4 expression was positively correlated with many drugs (BGB-283, TAK-632, MLN-2480, AZ-628, LXH-254, LY-3009120, Dabrafenib, Refametinib, Selumetinib, PLX-4720, and PLX-8394) and negatively correlated with others (GSK-60903, AZD-2858, Futibutinib, ON-123300, and LY-2090314). These results indicate that ETV4 expression could be used to guide the selection of chemotherapeutic agents.

Taken together, the present results demonstrate that ETV4 is upregulated in multiple cancers and that high expression of ETV4 is correlated with cancer progression and undesirable survival outcomes. Furthermore, ETV4 expression was correlated with immunity, tumor heterogeneity, MMR genes, DNA methyltransferase genes, and tumor stemness in multiple cancers. Finally, ETV4 expression was associated with drug sensitivity. Together these results suggest that ETV4 may be useful as a prognostic factor and therapeutic target for multiple cancers.

## Data availability statement

Publicly available datasets were analyzed in this study. This data can be found here: data for expression analysis were obtained from the UCSC database (https://xenabrowser.net/), TCGA Pan-Cancer (PANCAN,N=10535,G=60499) and TCGA TARGET GTEx (PANCAN,N=19131,G=60499). Any further inquiries can be directed to the corresponding author/s..

## Author contributions

NL and JH contributed to the study design and conception. RZ, KY, JQ, XW, WL, TJ, YZ, ZG, YP, DQ, and GW performed data analysis and wrote the main manuscript text. All authors contributed to the article and approved the submitted version.

## Supplementary material

Supplementary Figure 1 The relationship between ETV4 expression and three different immune scores in various cancers. **(A)** StromalScore, **(B)** ImmuneScore, **(C)** ESTIMATEScore.

Supplementary Table 1 The abbreviations of cancers.
