# Supplementary figures and images for: Oncogenic role and drug sensitivity of ETV4 in human tumors: a pan-cancer analysis
Source: Front Oncol. 2023 May 2;13:1121258. doi: 10.3389/fonc.2023.1121258 (PMC10185867; doi:10.3389/fonc.2023.1121258)

A

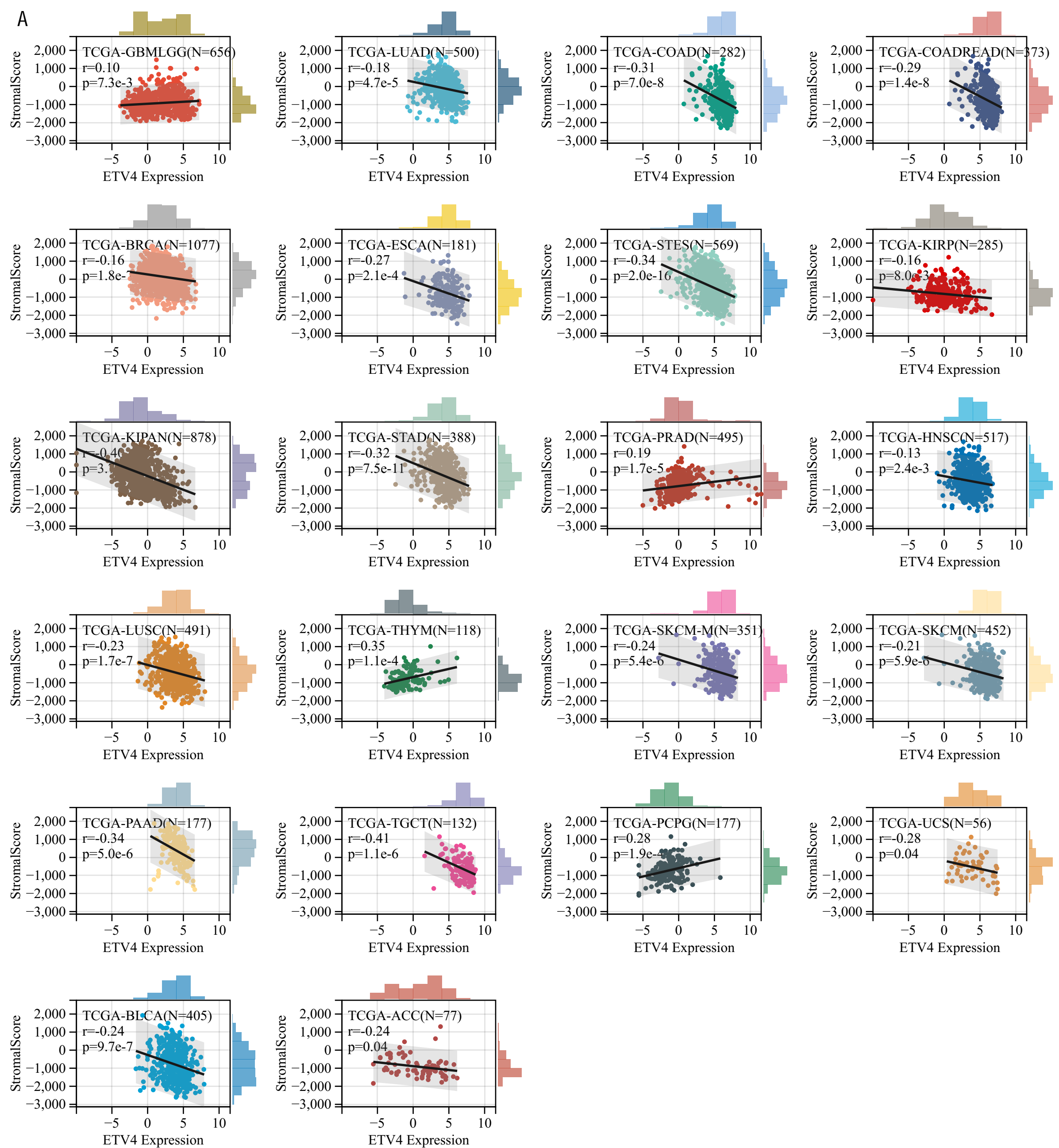

B

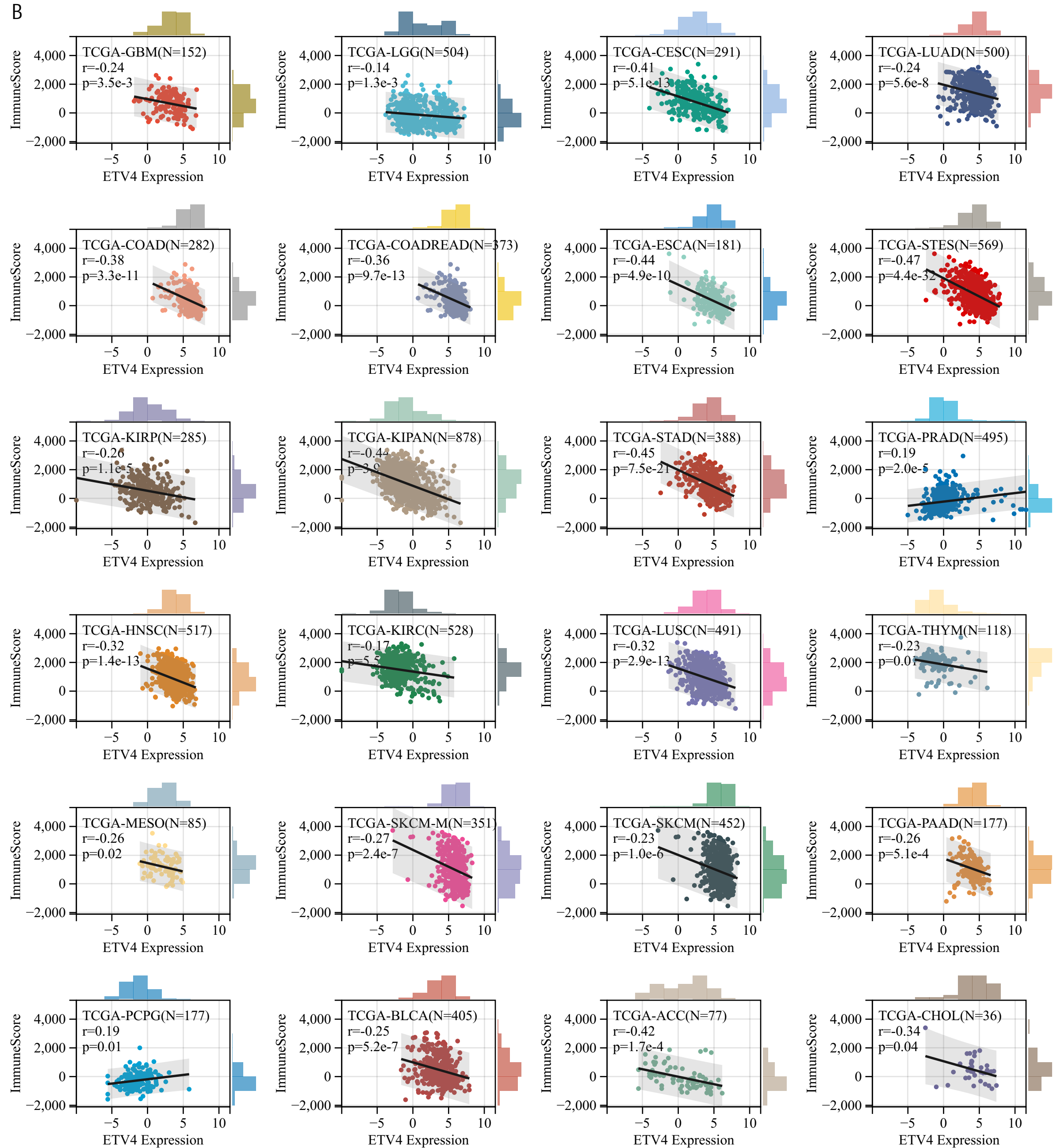

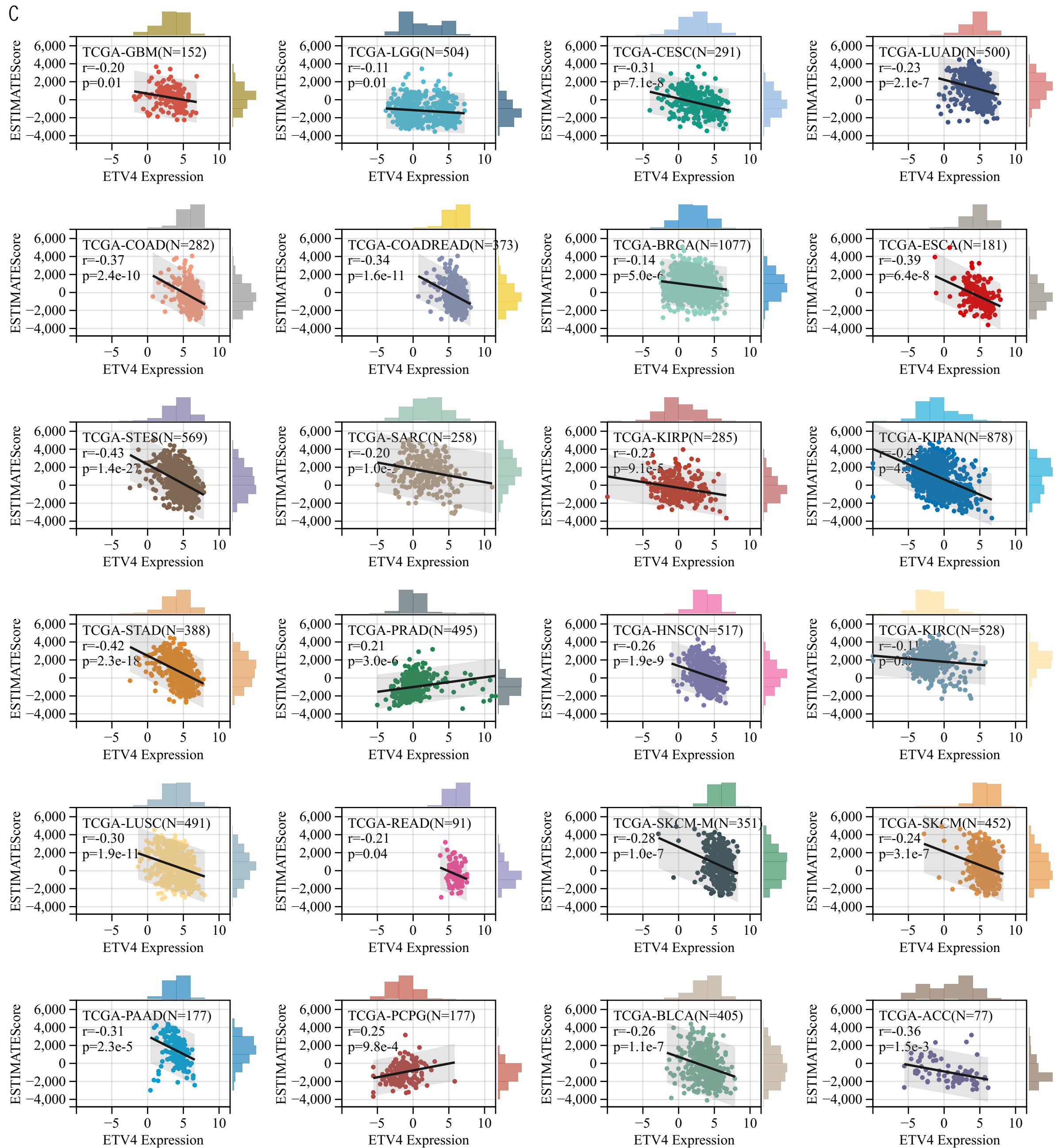

Supplement: Supplementary Figure 1 — The relationship between ETV4 expression and three different immune scores in various cancers. (A) StromalScore, (B) ImmuneScore, (C) ESTIMATEScore. [file Image_1.pdf]
