# Supplementary material for: Oncogenic role and drug sensitivity of ETV4 in human tumors: a pan-cancer analysis
Source: Front Oncol. 2023 May 2;13:1121258. doi: 10.3389/fonc.2023.1121258 (PMC10185867; doi:10.3389/fonc.2023.1121258)
Supplement: Supplementary Table 1 — The abbreviations of cancers. [file Table_1.pdf]

**Tumor name**	**abbreviation**	**Tumor name**	**abbreviation**
adrenocortical carcinoma	ACC	Liver hepatocellular carcinoma	LIHC
bladder Urothelial Carcinoma	BLCA	Lung adenocarcinoma	LUAD
breast invasive carcinoma	BRCA	Lung squamous cell carcinoma	LUSC
cervical squamous cell carcinoma and endocervical adenocarcinoma	CESC	Mesothelioma	MESO
cholangiocarcinoma	CHOL	Ovarian serous cystadenocarcinoma	OV
colon adenocarcinoma	COAD	Pancreatic adenocarcinoma	PAAD
Colon adenocarcinoma/Rectum adenocarcinoma Esophageal carcinoma	COADREAD	Pheochromocytoma and Paraganglioma	PCPG
Lymphoid Neoplasm Diffuse Large B-cell Lymphoma	DLBC	Prostate adenocarcinoma	PRAD
Esophageal carcinoma	ESCA	Rectum adenocarcinoma	READ
Glioblastoma multiforme	GBM	Sarcoma	SARC
Glioma	GBMLGG	Stomach adenocarcinoma	STAD
Head and Neck squamous cell carcinoma	HNSC	Skin Cutaneous Melanoma	SKCM
Kidney Chromophobe	KICH	Stomach and Esophageal carcinoma	STES
Pan-kidney cohort (KICH+KIRC+KIRP)	KIPAN	Testicular Germ Cell Tumors	TGCT
Kidney renal clear cell carcinoma	KIRC	Thyroid carcinoma	THCA
Kidney renal papillary cell carcinoma	KIRP	Thymoma	THYM
Acute Myeloid Leukemia	LAML	Uterine Corpus Endometrial Carcinoma	UCEC
Brain Lower Grade Glioma	LGG	Uterine Carcinosarcoma	UCS
Uveal Melanoma	UVM	Osteosarcoma	OS
Acute Lymphoblastic Leukemia	ALL	High-Risk Wilms Tumor	WT
